# Factors Influencing Timely Follow-up After Inconclusive Screening Mammograms at a 3D Mobile Mammography Center

**DOI:** 10.7759/cureus.69213

**Published:** 2024-09-11

**Authors:** Aditi Desai, Pura Rodriguez de la Vega, Grettel Castro, Prasad Bhoite, Julia Bisschops, Marcia Varella

**Affiliations:** 1 Department of Medical Education, Florida International University, Herbert Wertheim College of Medicine, Miami, USA; 2 Department of Humanities, Health, and Society, Florida International University, Herbert Wertheim College of Medicine, Miami, USA

**Keywords:** breast cancer, follow-up delays, inconclusive screening mammograms, mobile mammography, public health

## Abstract

Background

The Linda Fenner 3D Mobile Mammography Center (LFMMC) was created to reduce disparities in screening for breast cancer and improve survival for women with limited access to such screening. However, for screened patients requiring follow-up testing, research has shown a wide variation of time taken to access diagnostic imaging, especially among uninsured women.

Aim

To explore factors associated with longer (>30 days) time intervals to complete diagnostic imaging after screening.

Methods

This was a retrospective cohort study using data from the LFMMC from 2014 to 2022. Women living in Miami-Dade County (MDC), over the age of 40, uninsured, and requiring follow-up diagnostic imaging after breast cancer screening were included. Factors assessed included women’s age, area of residency, race, primary language spoken, marital status, body mass index (BMI), history of hormone use, previous mammogram, breast implants, breast cancer in immediate family, past breast surgery, and menopause status. The interval of time taken between receiving a screening mammogram result and completing further diagnostic imaging was the outcome, dichotomized into greater than 30 days (longer follow-up time) or up to 30 days. Multivariable binary logistic regression models were used to estimate independent associations (odds ratio [OR] and 95% confidence interval [CI]) of selected factors and longer time intervals to complete diagnostic imaging follow-up.

Results

We analyzed data from 926 eligible patients. The mean age was 50.1 years (SD = 8.81). A majority were white (n = 643; 69%), Hispanic (n = 698; 72%), had a history of previous mammograms (n = 589; 64%), and had no family history of breast cancer (n = 757; 82%). About 50% had a diagnostic follow-up exam performed after 30 days (median time to follow-up was 32, interquartile range 23-46 days). Single women had 30% lower odds of longer follow-up than married women (OR 0.70, 95% CI 0.52-0.93). Residents of Homestead (a city within Miami-Dade County) had 2.5 higher odds of having a longer time to follow-up, compared to non-Homestead residents (OR 2.50, 95% CI 1.60-3.90).

Conclusions

Being single was associated with a shorter time to follow up with diagnostic imaging while residing in Homestead was associated with a longer time to follow-up. Further research with a larger sample and further details on patient characteristics are warranted to better identify targets for interventions aimed at optimizing continuity of care and detecting breast cancer at earlier stages.

## Introduction

Breast cancer is the second most common cause of cancer-related deaths in women [1-3]. Screening mammograms have been shown to reduce breast cancer mortality rates and serve as an initial step to detect malignancies at an earlier stage [1,4,5]. Mammography screenings and subsequent timely treatment have prevented up to 614,000 deaths in United States (U.S.) women aged 40 to 84 between 1989 and 2018 [6]. However, uninsured and low-income women have significantly lower breast cancer screening rates compared to those who are insured and have a higher socioeconomic status [1,7,8]. In general, women living in high-poverty areas within the U.S. are 50% less likely to complete screening mammograms compared to those who live in higher socioeconomic areas [4].

Studies examining the characteristics of individuals being assessed at mobile mammography centers in the U.S. show that 48%-62% of the users are African American, 4-11% are Hispanic, and most have incomes below $25,000 and/or were uninsured [4]. This shows how mobile mammography centers are able to reach patient populations not commonly assessed in traditional mammography centers.

To improve access, the Linda Fenner 3D Mobile Mammography Center (LFMMC) was created in 2014. Since its inception, it has provided over 5,200 free screening mammograms within Miami-Dade County (MDC). Requirements for eligibility to be screened in the LFMMC include being a woman over the age of 40, uninsured, living within MDC, and having no current breast pain, lumps, history of breast cancer, or previous mammogram within the last year. The LFMMC visits different community partners at an average of 3 times per week to perform screenings at their site. The team is composed of a radiologist, breast health navigator, administrators, driver, technicians, and an overseeing physician. If a patient screened by the LFMMC requires follow-up imaging, the LFMMC utilizes a breast health navigator system. The navigator, together with the physician, helps patients understand mammogram results and guides them to appropriate diagnostic imaging outside of LFMMC. This service intends to help reduce barriers for women who require more testing. LFMMC services are funded by fundraising through the Florida International University (FIU) Mobile Mammography Initiative [9].

Since the inception of the LFMMC in 2014, 19.4% of all screened patients have required additional imaging after their screening mammogram on the LFMMC. Of note, the number of women needing additional follow-up has been increasing over the years. In the most recent assessment, the need for follow-up of screening mammograms has been as high as 35% [10]. The LFMMC benchmark for time taken between receiving an inconclusive LFMMC screening and completing further imaging is 30 days. Findings from a mobile mammography van primarily serving uninsured patients within the U.S. suggest that patients on the mobile center were approximately twice as likely to experience delays in follow-up compared to patients from a fixed clinic [11]. Other mobile centers have reported benchmarks of 60-90 days [12,13], with patients experiencing even longer follow-up delays [11,14].

Overall, there is a limited amount of literature on what factors are associated with timely follow-up after screenings. Some cited reasons for this variability include sociodemographic factors (age and race), differences in Breast-Imaging Reporting and Data System (BI-RADS) scores, and miscommunication of screening results [11,14-16]. Qualitative and quantitative studies investigated the reasons for delay in follow-up care. For instance, a study assessing abnormal screening results (BI-RADS 0, 4, and 5) from 317 women aged 33-85 years old within a Northern California mobile van program found that, after adjusting for age, family history, and income, non-white women experienced an average of 15 days for follow-up imaging while white women experienced an average of 7 days (p <0.001) [16]. Additionally, analyses of data for 636 women with abnormal screening results (BI-RADS 0, 3, 4, and 5) from a Cook County mobile mammography service showed that patients with a BI-RADS of 4 were 3.5 times more likely to have timely follow-up as compared to those with BI-RADS 0 (95% confidence interval [CI]: 1.46-8.61) [14]. These findings suggest a role for race/ethnicity and having abnormal/inconclusive screening results on time to complete a follow-up breast imaging after the initial screening. However, these studies were published more than a decade ago and changes in health care might have occurred since then, thus, findings require an updated assessment. In 2022, abnormal radiographic results (BI-RADS 0) from 1,337 women in either a mobile mammography service (MM) or fixed clinic were assessed for the potential association between sociodemographic/medical factors and time to follow-up. Only 45% of MM patients obtained follow-up within 60 days, as compared to 72% of fixed-site patients (p<0.001) Furthermore, African American women (95% CI: 1.0-2.1) were more likely to experience delays. Married women (95% CI: 0.5-0.9), those with a previous history of breast cancer (95% CI: 0.2-0.8) or family history (95% CI: 0.6-0.9) were less likely to experience delays [11].

Based on these findings, we aimed to explore select factors that could be associated with a longer (>30 days) time interval in completing diagnostic imaging within previously screened LFMMC patients who require diagnostic follow-up exams.

This article was previously presented as a poster at the 2024 National Consortium Breast Centers Conference on March 16, 2024. 

## Materials and methods

Study design and participants

This was a retrospective cohort study using data from the LFMMC. To be screened through LFMMC, women had to live in MDC, be over the age of 40, be uninsured, not be experiencing breast pain and/or lumps, have no history of breast cancer, and have not had a previous mammogram in the past year. A total of 1296 eligible LFMMC patients who received an inconclusive screening mammogram between 2014-2022 and required follow-up diagnostic imaging were identified.

Variables

The sociodemographic variables assessed included the participant’s primary language spoken, age, city of current MDC residence, race, ethnicity, and current marital status. The variable “cities” included Homestead, Hialeah, Opa Locka, Miami Gardens, Miami, Miami Beach, North Miami Beach, and Key Biscayne. Each city was grouped using established ZIP codes [17]. Clinical variables assessed included the participant’s body mass index (BMI) at the time of visit, history of any prior mammogram completion, family history of breast cancer, history of breast implants, past breast surgery (including biopsy, breast reduction, or cyst aspiration), menopausal status, and current hormone use. The dependent variable assessed the interval of time taken between receiving a screening mammogram and completing further diagnostic imaging, dichotomized into greater than 30 days (longer follow-up time) or up to 30 days. Dependent variables were retrieved through medical records.

Statistical analysis

Characteristics of participants were initially described. Summary measures included mean/median (standard deviations and interquartile ranges) for continuous variables and proportions for categorical variables. The outcome was dichotomized by less than or equal to 30 days and more than 30 days, and the distribution of the variables was compared according to the outcome levels. A benchmark of 30 days was utilized based on internal quality control decisions by the LFMMC clinical team. Chi-square tests were used to test for significant differences within categorical variables. Mann-Whitney test and Kruskal Wallis test were used to compare the median time interval according to selected characteristics. The correlation matrix of all factors included in the model was examined to check for collinearity. Lastly, the association between the covariates and the outcome (time taken to follow-up imaging) was assessed using binary logistic regression models. Odds ratios (OR) and corresponding 95% CI were estimated. Effect modification was assessed using interaction terms in the regression models with a plan to perform stratified analyses if interactions were found. Significance was considered for p-values less than or equal to 0.05 (two-tailed test). Stata software version 15.0 (StataCorp, College Station, USA) was used for all analyses. The Florida International University Health Sciences Institutional Review Board approved this study (Approval Number: IRB-17-0142-AM05, Reference Number: 105438).

## Results

Total number of eligible women was 1,296. Of this, 370 women (29%) were excluded due to missing data. Reasons for this missing data include patient variability in the completion of demographic and clinical intake forms and changes made to forms. Therefore, 926 participants were evaluated in this study.

The mean age was 50.1 years (SD = 8.8), with more than half of the sample (n = 554; 59.8%) within the 38-49 years age range (Table 1). A majority were White (n = 643; 69.4%), Hispanic (n = 698; 71.5%), and Spanish-speaking (n = 586; 63.3%). Most were either married (n = 364; 39.3%) or single (n= 381; 41.1%). Within MDC, the participants mostly commonly resided in Miami (n = 674; 72.8%), followed by Homestead (n = 111; 12.0%), Hialeah (n = 74; 8.0%), Miami Beach (n = 28; 3.0%), Opa Locka (n = 14; 1.5%), North Miami Beach (n = 11; 1.2%), Miami Gardens (n = 10; 1.1%), and Key Biscayne (n = 3; 0.3%). A majority of the sample had a history of previous mammograms (n = 589; 63.6%), no family history of breast cancer (n = 757; 81.8%), no breast implants (n = 887; 95.8%), no history of hormone use (n = 898; 97%), and no history of breast cancer surgery or biopsy (n = 839; 90.6%).

**Table 1:** Characteristics of Linda Fenner 3D Mobile Mammography Center patients who underwent a screening mammogram follow-up N: Number of participants, BMI: Body Mass Index ^1^History of breast cancer surgeries, biopsy, breast reduction, or cyst aspiration

Characteristics	N (%)
Age (years)	
38-49	554 (59.8)
50-59	200 (21.6)
60 or older	172 (18.6)
Race	
White	643 (69.4)
Black	283 (30.6)
Ethnicity	
Non-Hispanic White	24 (2.5)
Non-Hispanic Black	248 (25.4)
Hispanic	698 (71.5)
Other Non-Hispanic	6 (0.6)
Marital Status	
Married	364 (39.3)
Divorced/Separated	139 (15.0)
Single	381 (41.1)
Widowed	42 (4.5)
Cities	
Homestead	111 (12.0)
Hialeah	74 (8.0)
Opa Locka	14 (1.5)
Miami Gardens	10 (1.1)
Miami	674 (72.8)
Miami Beach	28 (3.0)
North Miami Beach	11 (1.2)
Key Biscayne	3 (0.3)
Outside Miami-Dade	1 (0.1)
Language	
English	225 (24.3)
Creole	115 (12.4)
Spanish	586 (63.3)
BMI	
Underweight	5 (0.5)
Normal Weight	198 (21.4)
Overweight	349 (37.7)
Obese	374 (40.4)
Previous Mammogram	
No	337 (36.4)
Yes	589 (63.6)
Family History of Breast Cancer	
No	757 (81.8)
Yes	169 (18.3)
Breast Implants	
No	887 (95.8)
Yes	39 (4.2)
Past Surgery^1^	
No	839 (90.6)
Yes	87 (9.4)
Menopausal Status	
Premenopause	509 (55.0)
Menopause	417 (45.0)
Current Hormone Use	
No	898 (97.0)
Yes	28 (3.0)

**Figure 1:**
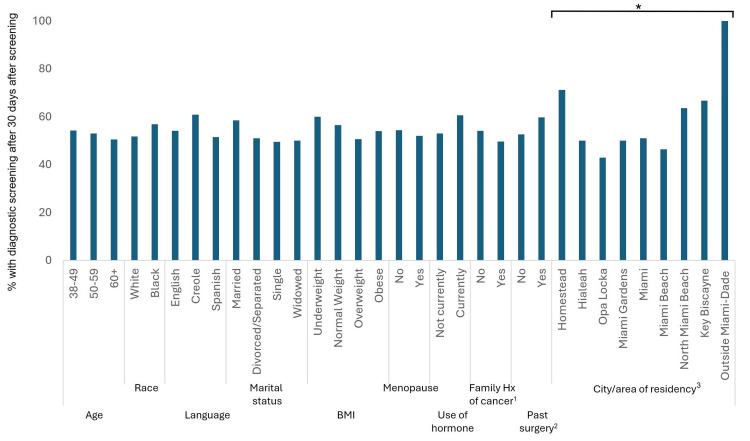
Frequency of Linda Fenner 3D Mobile Mammography Center patients undergoing diagnostic mammogram follow-up after the 30-days benchmark by selected characteristics BMI: Body Mass Index, Hx: History *p-value = 0.0081; All p-values were calculated using Chi-square test unless otherwise specified. ^1^Family history of breast cancer ^2^History of breast cancer surgeries, biopsy, breast reduction, or cyst aspiration ^3^Fisher’s Exact Test

The median time to follow-up within our patient sample was 32 days and the interquartile range was 22.5 days to 46 days. Time to diagnostic mammogram was split into less than or equal to 30 days, and more than 30 days. About 50% had a diagnostic follow-up exam performed after 30 days. Overall, there was no difference in the frequency of time to a follow-up diagnostic mammogram for most of the patient characteristics (Figure 1). Patients residing in Homestead (71.2%) more frequently had a longer time to follow-up compared to other cities within MDC (46.4% for Miami Beach).

Patients residing in Homestead had one of the highest median intervals of time from screening to diagnostic exams (41 days, interquartile range 29-54 days) and single women had one of the lowest intervals (30 days, interquartile range 22-44 days; Table 2).

**Table 2:** Median number of days from screening to diagnostic mammogram to follow-up based by selected characteristics IQR: Interquartile range, BMI: Body Mass Index *p <0.05 ^1^P-values were calculated using non-parametric tests (either Mann-Whitney or Kruskal-Wallis test) due to the non-normal distribution of the number of days from screening to diagnostic imaging. ^2^History of breast cancer surgeries, biopsy, breast reduction, or cyst aspiration

Characteristics	Median (IQR)	p-value^1^
Age (years)		0.934
38-49	32 (22-45)	
50-59	32 (23.5-46)	
60+	31 (22-49)	
Race		0.112
White	31 (23-44)	
Black	34 (22-51)	
Marital Status		0.488
Married	34 (24-47.5)	
Divorced/Separated	31 (22-49)	
Single	30 (22-44)	
Widowed	31 (21-43)	
Cities		0.002*
Homestead	41 (29-54)	
Hialeah	30 (21-43)	
Opa Locka	23.5 (21-42)	
Miami Gardens	30.5 (19-40)	
Miami	31 (22-44)	
Miami Beach	29.5 (25.5-37.5)	
North Miami Beach	34 (15-43)	
Key Biscayne	35 (14-40)	
Outside Miami-Dade Co	65 ( - )	
BMI		0.302
Underweight	42 (21-62)	
Normal Weight	32 (24-51)	
Overweight	31 (22-43)	
Obese	32 (22-47)	
Previous Mammogram		0.571
No	32 (23-44)	
Yes	32 (22-48)	
Family History of Breast Cancer		0.658
No	32 (23-46)	
Yes	30 (22-48)	
Breast Implants		0.471
No	32 (23-46)	
Yes	29 (22-44)	
Past Surgery^2^		0.230
No	32 (22-45)	
Yes	33 (23-54)	

Only two factors were found to be associated with the time interval from screening to diagnostic testing. In the unadjusted model, the odds of having a follow-up time greater than 30 days decreased by 30% in single patients (OR = 0.70, 95% CI 0.52-0.93), compared to married participants. After adjusting for confounding variables, the difference remained (OR = 0.70, 95% CI 0.52-0.95; Table 3, adjusted model 2). Patients residing in Homestead were 2.5 times more likely to have a longer time to follow-up, compared to residents in other MDC areas (OR 2.50, 95% CI 1.60-3.90; Table 3, adjusted model 2). No other association was significant in the adjusted model.

**Table 3:** Associations between selected patients’ characteristics and follow-up with diagnostic exam after 30 days from breast cancer screening BMI: Body Mass Index, OR: Odds Ratio *The model was adjusted for select variables, based on collinearity assessment. Adjusted model 2 includes the addition of cities. **p <0.05 ^1^All p-values were calculated using binary logistic regression. ^2^History of breast cancer surgeries, biopsy, breast reduction, or cyst aspiration

Characteristics	Unadjusted		Adjusted Model 1*		Adjusted Model 2*	
	OR (95% CI)	p-value^1^	OR (95% CI)	p-value	OR (95% CI)	p-value
Age (years)						
38-49	reference	-	reference	-	reference	-
50-59	0.95 (0.69 -1.31)	0.746	0.92 (0.65-1.30)	0.638	0.88 (0.62-1.25)	0.464
60+	0.86 (0.61-1.21)	0.389	0.82 (0.56-1.18)	0.280	0.80 (0.55-1.16)	0.236
Race						
White	reference	-	reference	-	reference	-
Black	1.23 (0.93-1.63)	0.152	1.25 (0.93-1.67)	0.140	1.35 (0.99-1.83)	0.057
Marital Status						
Married	reference	-	reference	-	reference	-
Divorced/Separated	0.79 (0.50-1.10)	0.133	0.79 (0.53-1.18)	0.256	0.83 (0.55-1.26)	0.382
Single	0.70 (0.52-0.93)	0.015	0.69 (0.51-0.92)	0.012	0.70 (0.52-0.95)	0.021**
Widowed	0.71 (0.37-1.34)	0.292	0.73 (0.38-1.42)	0.355	0.71 (0.36-1.39)	0.312
Cities						
Homestead	2.37 (1.53-3.67)	0.00	-	-	2.50 (1.60-3.90)	0.000**
Hialeah	0.96 (0.59-1.55)	0.865	-	-	1.09 (0.66-1.80)	0.735
Opa Locka	0.72 (0.25-2.10)	0.546	-	-	0.68 (0.23-2.01)	0.489
Miami Gardens	0.96 (0.28-3.34)	0.948	-	-	0.86 (0.24-3.04)	0.812
Miami Beach	0.83 (0.39-1.77)	0.633	-	-	0.92 (0.42-2.01)	0.834
North Miami Beach	1.68 (0.49-5.79)	0.412	-	-	1.76 (0.50-6.23)	0.377
Language						
English	reference	-	-	-	-	-
Creole	1.31 (0.83-2.07)	0.243	-	-	-	-
Spanish	0.90 (0.66-1.22)	0.493	-	-	-	-
BMI						
Normal Weight	reference	-	reference	-	reference	-
Overweight	0.79 (0.56-1.12)	0.188	0.76 (0.53-1.09)	0.135	0.73 (0.51-1.06)	0.100
Obese	0.90 (0.64-1.28)	0.559	0.88 (0.62-1.27)	0.514	0.89 (0.62-1.28)	0.524
Previous Mammogram						
No	reference	-	reference	-	reference	-
Yes	1.05 (0.81-1.38)	0.703	1.09 (0.82-1.47)	0.547	1.08 (0.80-1.46)	0.596
Family History of Breast Cancer						
No	reference	-	reference	-	reference	-
Yes	0.84 (0.60-1.17)	0.294	0.87 (0.61-1.23)	0.424	0.87 (0.61-1.24)	0.434
Breast Implants						
No	reference	-	reference	-	reference	-
Yes	0.74 (0.39-1.41)	0.359	0.65 (0.33-1.28)	0.213	0.64 (0.32-1.27)	0.200
Past Surgery^2^						
No	reference	-	reference	-	reference	-
Yes	1.33 (0.85-2.09)	0.208	1.33 (0.83-2.11)	0.233	1.33 (0.83-2.12)	0.239
Menopausal Status						
Premenopause	reference	-	-	-	-	-
Menopause	0.91 (0.70-1.18)	0.470	-	-	-	-
Current Hormone Use						
No	reference	-	reference	-	reference	-
Yes	1.36 (0.63-2.94)	0.429	1.36 (0.62-2.99)	0.438	1.23 (0.55-2.74)	0.607

## Discussion

Our findings identified single marital status to be associated with odds of shorter time to follow-up and residence in Homestead with odds of longer time to follow-up compared to their counterparts.

For marital status, single women might have greater flexibility in time to complete screenings compared to married women. The responsibilities of married women within their families might limit their access to health care. However, our findings are contrary to those of a study of 1,337 women in New York City. In this study, the odds of a longer time to follow-up were 37% less in married women compared to unmarried women while accounting for race, ethnicity, and family history of breast cancer [11]. Of note, our study sample consists of a unique patient demographic, characterized by uninsured women in MDC that only utilized a mobile mammography service. Furthermore, the cutoff interval to consider delayed care varied from 30 days in our study, as opposed to 60 days in the New York study. We also performed analyses using the cut-off of 60 days to define our study outcome (data not shown). Using the 60 days cut-off, results were not significant for all variables assessed, likely due to the very small numbers of women in our sample having completed the diagnostic exam after 60 days. Despite the inconsistency of results that requires confirmation, the results are not implausible. Further research should confirm these findings and investigate how marital status within an uninsured patient population plays a role in access to care.

Patients residing in Homestead had an increase in odds of longer time to follow-up compared to patients residing in other areas of MDC. There is limited literature on the differences in healthcare access within cities of MDC. The LFMMC visits various community partners within MDC to perform breast cancer screenings. However, differences in time to follow up within Homestead could potentially point towards the need for tailored attention to certain areas of residence. Further research is needed to confirm such findings, and if so, to assess and propose strategies aimed at greater accessibility to diagnostic imaging sites and decreasing patient-specific barriers within the Homestead region.

Our results for race did not reach statistical significance, possibly due to limitations in the sample size. For Black women, the odds for longer follow-up were 1.35 compared to whites (95% CI: 0.99-1.83). Thus, there is a possibility that the odds of delayed follow-up were either not present or up to 83% higher, which would suggest a non-negligible difference. Consistent with that, results of a 1993-1994 study of women from Northern California suggested that non-white women had a median time to follow-up of 19 days higher than white women (average 12 days) even after accounting for age, family history, palpable mass, and income [16]. 

Lastly, previous studies have shown that in women with a family history of breast or ovarian cancer, the odds of delayed diagnostic exams were 24% lower [11]. We did not find such an association between a family history of breast cancer and delayed access to diagnostic exams. The present study was likely underpowered to test such an association. As we increase the number of participants being screened at the LFMMC, further analyses should be conducted.

There are certain limitations to our study that need to be discussed. We performed secondary analyses of data that was collected unsystematically and for clinical purposes. All patients included in our sample had a BI-RADS score registered as “0”. Thus, a BI-RADS screening score of 4 or 5, which would indicate possible/likely malignancy, could lead to a shorter time to follow-up compared to those with a BI-RADS score of 3. Such hypotheses could not be tested in our study. Additionally, information on factors such as a patient’s history of disabilities, health literacy, and family income were not available to be assessed as confounders. It is likely that patients with low income, low health literacy, or disability might be at a greater risk for lack of access to screening mammograms and therefore experience a longer time to follow-up [7,18,19]. Such information would help in improving care for these patient populations. Also, information on the dates of previous mammogram screenings performed by the patient (to allow assessment of adherence to biennial screening guidelines) were not available. We believe that further research should investigate the relationship between adherence to screening guidelines and time to follow-up. A longer gap in screenings could impact the level of comfort and motivation in completing further diagnostic mammograms. Additionally, the healthcare characteristics of LFMMC might differ from other local mobile mammography services, limiting external validity. Qualitative differences in the program structure and members of a team all play a role in ultimately guiding the patient to diagnostic imaging completion [20,21]. However, considering the scarcity of information available, we believe that this study provides insight to inform similar outreach healthcare programs. 

## Conclusions

LFMMC offers screening services within MDC and allows us to assess patterns of screening within a unique patient population. Overall, the time taken to complete diagnostic imaging after screening mammograms for select patients is variable.

Our study showed that unmarried women had lower odds of taking a longer time to follow up with diagnostic mammography after an inconclusive screening result, compared to married patients. Patients residing in Homestead had higher odds of delay in follow-up compared to other areas of MDC. Future research should continue to identify potential targets for interventions aimed at optimizing the early detection of breast cancer and ultimately improving women’s health in an equitable way.
